# Stress and Alterations in Bones: An Interdisciplinary Perspective

**DOI:** 10.3389/fendo.2017.00096

**Published:** 2017-05-01

**Authors:** Pia-Maria Wippert, Michael Rector, Gisela Kuhn, Karin Wuertz-Kozak

**Affiliations:** ^1^Department of Health Sciences, Institute of Sociology of Health and Physical Activity, University of Potsdam, Potsdam, Germany; ^2^Department of Health Sciences and Technology, Institute for Biomechanics, ETH Zurich, Zurich, Switzerland; ^3^Schön Klinik München Harlaching, Munich, Germany; ^4^Spine Center, Academic Teaching Hospital and Spine Research Institute, Paracelsus Private Medical University Salzburg, Salzburg, Austria; ^5^Competence Center for Applied Biotechnology and Molecular Medicine (CABMM), University of Zurich, Zurich, Switzerland

**Keywords:** biomechanics, bone–brain–nervous system interactions, endocrine pathways, osteoporosis, exercise

## Abstract

Decades of research have demonstrated that physical stress (PS) stimulates bone remodeling and affects bone structure and function through complex mechanotransduction mechanisms. Recent research has laid ground to the hypothesis that mental stress (MS) also influences bone biology, eventually leading to osteoporosis and increased bone fracture risk. These effects are likely exerted by modulation of hypothalamic–pituitary–adrenal axis activity, resulting in an altered release of growth hormones, glucocorticoids and cytokines, as demonstrated in human and animal studies. Furthermore, molecular cross talk between mental and PS is thought to exist, with either synergistic or preventative effects on bone disease progression depending on the characteristics of the applied stressor. This mini review will explain the emerging concept of MS as an important player in bone adaptation and its potential cross talk with PS by summarizing the current state of knowledge, highlighting newly evolving notions (such as intergenerational transmission of stress and its epigenetic modifications affecting bone) and proposing new research directions.

## Introduction

Bones are an essential component of the musculoskeletal system, with their primary functions being protecting vital organs, supporting the body, assisting in movement, producing blood cells, and storing nutrients and minerals. To fulfill all this functions, bone mass and structure are regulated by a number of factors. Bone tissue is continuously remodeled and modeled to maintain a healthy matrix and to adapt to changing environmental factors. Disturbances in these mechanisms often result in reduced bone mass and an increased risk for fractures, with aging (especially in postmenopausal women), furthermore, impacting bone health. Natural aging leads to the accumulation of osteoporosis risk factors, including a gradual inability to cope with physical and mental stressors (with consequences on bone adaptation) as well as biochemical alterations elucidated in more detail within this perspective.

## Physical Stress (PS): A Brief Summary

Mechanical loading, also often termed as PS, is an important trigger inducing structural adaptation in bone. Multitudes of studies have investigated the effects of PS on bone, including the identification of loading regimes supporting healthy tissue homeostasis (or reversely degeneration-associated matrix loss), analysis of underlying mechanotransduction mechanisms, as well as elucidation of load-induced molecular responses (1–5). Nowadays, it is widely accepted that osteocytes are the main sensors of mechanical forces and orchestrate the activity of osteoblasts (responsible for bone formation) and osteoclasts (responsible for bone resorption) by several signaling pathways (6).

## Mental Stress (MS): A Brief Summary

**Figure 1:**
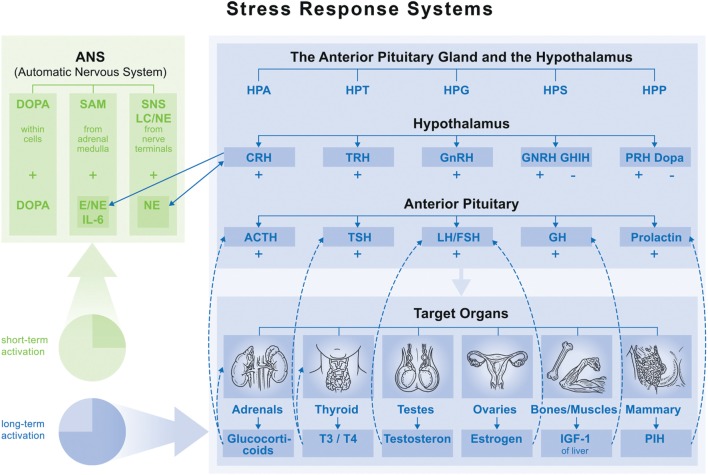
**Overview of main endocrine axes**. Normal function: feedback loop stops activation on each axis. Maladaptation of feedback function leads to fluctuation (accumulation) in one axis (hyper- or hypofunction), which influences function of the others. ⇢ = inhibition; → = stimulation. Abbreviations: HPA, hypothalamic–pituitary–adrenal; HPT, hypothalamic–pituitary–thyroid; HPG, hypothalamic–pituitary–gonadal; HPS, hypothalamic–pituitary–somatotropic; HPP, hypothalamic–pituitary–prolactin; CRH, corticotropin-releasing hormone; TRH, thyrotropin-releasing hormone; GnRH, gonadotropin-releasing hormone; GHRH, growth hormone-releasing hormone; GHIH, growth hormone-inhibiting hormone; DOPA, dopamine; ACTH, adrenocorticotropic hormone; TSH, thyroid-stimulating hormone; LH, luteinizing hormone; FSH, follicle-stimulating hormone; GH, growth hormone.

Recent publications provide compelling evidence that psychosocial stress, defined here as MS, can disturb bone homeostasis. When studying signaling pathways and pathological consequences of MS different stressor characteristics are distinguished. Till now it is well known that specific social stress situations (e.g., social evaluative or threat components) provoke the strongest physiological responses (7). However, individuals respond differently to these situations depending on their interpretation, resources, and adaptation strategies, which refer also to their biographical time frames and the duration of the stress exposition (short term, such as acute or long term such as chronic, traumatic stress type). Thus, an understanding of physiological mechanisms behind the MS response is particularly complex (see Figure 1) (8). Biologically, both responses (short-/long-term MS) are driven by the autonomic nervous system and the hypothalamic–pituitary axis. Within the autonomic nervous system, the stress response proceeds to one of three peripheral catecholamine systems (sympathetic–nervous system, sympathetic–adrenal–medullary system, and dopamine systems), whereby their operation depends on stressor type and characteristic (9). In the anterior pituitary, the stress response is determined by hypothalamic nuclei interactions and neuroendocrine cell hormone regulation. It is influenced by the stressor characteristic, driven by synaptic input from different brain regions like the limbic system (hippocampus, amygdala), as well as the brainstem (locus coeruleus), and realized on five endocrine axes: hypothalamic–pituitary–adrenal (HPA), hypothalamic–pituitary–thyroid (HPT), hypothalamic–pituitary–gonadal (HPG), hypothalamic–pituitary–somatotropic (HPS), and hypothalamic–pituitary–prolactin (HPP).

While these systems promote adaptation and allostasis (i.e., active maintenance of homeostasis) for performance in challenging situations, recent research shows that their repeated activation accumulates allostatic load leading to allostatic overload. This results in long-term maladaptation, which is often described in an HPA hyper- or hypofunction which then invokes immune, cardiovascular, or metabolic system maladaptation. The development of allostatic load is based on neural, neuroendocrine, and neuroendocrine-immune mechanisms and associated with a long list of pathologies [HPA hyperfunction: e.g., depression, Cushing’s syndrome, and type II diabetes; HPA hypofunction: e.g., multiple sclerosis and rheumatoid arthritis (8, 10)].

## MS: Effects on Bone Structure and Function

Although the exact mechanisms remain to be elucidated and potential confounders (e.g., comorbidities, pharmacological therapies, physical inactivity, and sex) to be excluded, depression and anxiety have more recently been identified as additional risk factors for disturbed bone homeostasis, osteoporosis, and fractures in humans (11–14). For example, postmenopausal women suffering from depression exhibited decreased lumbar vertebra and femur DEXA scores compared to non-depressed controls, indicating a possible relationship between MS and bone mineral density (BMD) (15). A negative association between depression and BMD has been found in the majority of studies for both sexes, although many studies have not clearly defined MS (11, 13). Unfortunately, results on MS and BMD are often obtained from studies focusing on other topics (e.g., obesity/diabetes) and, furthermore, do not provide information discerning between acute or traumatic episodic stress effects. Therefore, future research in humans should differentiate specific stressor types and characteristics and improve patient selection and confounder controlling (smoking, medication, and physical activity) in studies. A systematical evaluation of pathways and mechanism linking MS and bone health (structure/function) could help establishing a conceptual framework to estimate whether stress-related effects will be naturally compensated for or can be blunted or even reversed naturally or therapeutically.

Compared to human studies, animal models allow for tighter control of experimental conditions, avoidance of confounders, and lower subject variability. A variety of methods exist to induce stress in animals, and these are often combined to complex regimes to prevent habituation effects: water deprivation, overnight illumination, stroboscopic illumination, cage tilt, housing in soiled cages, or exposure to noise (16, 17). Traumatic stress can be simulated by application of electric (foot) shocks (18, 19), exposure to predators (20) or physical restraint (21), or by exposure of pups to unpredictable maternal separation (22). Aside from selecting a suitable stress model to test the respective study hypothesis, choosing the most appropriate species is also crucial. Historically, a variety of animals have been used in bone research, but species differences, e.g., lack of a trabecular and Haversian remodeling in rodents, exaggerates the importance when choosing the most appropriate model (23–27). In rats, MS promotes disintegration of periodontal bone tissue (28). In mice, exposure to chronic stress activates bone resorption and suppresses bone formation shown by reduced BMD, deteriorated microarchitecture and/or altered biochemical markers (18, 29, 30). Further studies have investigated the role of stressor characteristics (social isolation/electroshock) on bone mass in mice, concluding that bone mass can be affected by different stressor characteristics (31). Future research would benefit from using newer techniques, such as *in vivo* micro-computer tomography imaging, to monitor changes in bone microarchitecture throughout an experiment (32, 33). Compelling evidence indicates early life stress has epigenetic consequences in humans and animals with similar findings emerging for chronic stress and depression in adults (34–38). Therefore, focusing on animal models would be useful in studying the consequences of intergenerational MS transmission on bone biology as well as the testing of therapeutic interventions, pharmaceutical as well as environmental (e.g., enriched living conditions) (39).

## MS: Molecular Mechanism Leading to Bone Adaptation

Currently, the key molecular mediators between MS and bone health are considered to be growth hormones (GH), glucocorticoids, and inflammatory cytokines (see Figure 1). Levels of GH are altered by MS through modulation of the HPA axis and consequent upregulation of growth hormone-releasing hormone and growth hormone-inhibiting hormone (40). Recent *in vitro* studies demonstrated that GH can directly induce osteoblast proliferation and differentiation (41–43). For example, in an embryonal rat tibiae model, GH not only increased alkaline phosphatase levels but also induced local production of insulin-like growth factor-I (IGF-I) (41). In fact, numerous other studies have described the stimulatory effect of GH on IGF-I production and its role as an important growth factor in bone (44–48). Using IGF-I-overexpressing mice, Zhao et al. were able to demonstrate that IGF-I has anabolic effects by primarily promoting the activity of resident osteoblasts, but potentially also by prolonging the life span of osteocytes (46). Circulating IGF-I is then detected by the hypothalamus and pituitary gland, completing the negative feedback loop for gaining allostasis through suppressing GH secretion. When this feedback system is desensitized through chronic stress, GH deficiency can develop leading to catabolic effects on bone (49, 50). Mouse models of IGF-I and GH deficiencies present with (up to 87%) reduced postnatal bone mineral content (51), as well as (approximately 40%) reduced femoral length and bone size (52). Among other mechanisms, these outcomes are related to the fact that parathyroid hormone (PTH), which has well-known anabolic, bone-forming effects, is dependent on osteoblast-driven IGF-I production (53). Similarly, humans with GH deficiency acquired in adulthood show decreased BMD (54, 55). Furthermore, aging diminishes GH/IGF-I secretion and response and causes gradual deterioration of both the immune and endocrine systems (56).

The HPA axis is, furthermore, responsible for the release of glucocorticoids (predominately cortisol) (40), which in turn influences the basal HPA activity and termination of the stress response by acting on other regulatory centers, such as the hippocampus, frontal cortex, hypothalamus, and the pituitary gland (40, 57). Although exact mechanisms are not completely understood, glucocorticoids are believed to have a multifaceted and dose-dependent role in bone formation and homeostasis. At physiological levels, glucocorticoids promote bone formation through induction of osteogenic differentiation of progenitor cells (58). In contrast, elevated levels of cortisol directly inhibit osteoblast proliferation, differentiation, and apoptosis in various species (59–62), which could substantially blunt the bone formation process leading to lower bone density. Importantly, aging causes greater activation of the HPA axis, which results in elevated production of glucocorticoids and stronger feelings of stress, anxiousness, and depression as well as an overall detrimental shift (63).

Aside from direct effects on bone cells, glucocorticoids also inhibit GH and gonadal steroid production, further reducing bone mass. The effects of high cortisol levels are seen in patients suffering from Cushing’s syndrome, which typically present with decreased bone mass and quality (64, 65) as well as in certain types of depression and chronic anxiety disorders (see Mental Stress (MS): A Brief Summary) (16, 66–68).

Glucocorticoids further influence the transport and function of leukocytes and thus inhibit the production of pro-inflammatory cytokines [e.g., tumor necrosis factor alpha (TNF-α), interleukin (IL)-1β, and IL-6], e.g., *via* glucocorticoid receptor (GR)-induced suppression of nuclear factor kappa B and activator protein 1 (69). While these findings suggest an overall anti-inflammatory effect of stress, *in vivo* mechanisms are more complex. Recent research demonstrates chronic stress can induce GR resistance leading to decreased sensitivity of immune cells to glucocorticoids and a resultant inability to downregulate inflammatory responses (50, 70). GR resistance and impaired HPA responsiveness—relevant in numerous inflammatory diseases (e.g., rheumatoid arthritis)—play supposedly a role in the development of osteoporosis (71). Interestingly, patients suffering from chronic inflammatory diseases have a higher prevalence of osteoporotic fractures, providing an additional indirect link between GR resistance and bone pathologies (72). Aside from GR resistance, corticotropin-releasing hormone (CRH), also secreted during MS, induces the release of IL-6 (73–75). High levels of IL-6 and other cytokines, such as IL-1β and TNF-α, affect differentiation of mesenchymal stem cells, suppress osteoblast function, initiate osteoclastogenesis, and activate osteoclast function (76, 77). These findings indicate a delicate inflammatory balance exists to ensure appropriate bone formation *in vivo*.

Although the isolated pathways of these three mediators of bone mass are well evaluated, knowledge regarding their interaction and buffering effects due to environmental conditions in enriched living conditions is lacking. For this reason, future research should include genetically modified animals, such as conditional IGF-I knockout mice (78), opening the possibility for mechanistic investigations as well as environmental mediators like PS.

## Interplay Between MS and PS

Despite no studies having been conducted to directly investigate the interplay between PS and MS, indirect indications of potential cross talk exist. Studies examining the loading of bone cells under different biochemical conditions (MS conditions or normal culture conditions) and/or with subsequent analysis of MS biomarkers have provided useful data.

One molecule that may allow interplay between stress types is IGF-I (see MS: Effects on Bone Structure and Function). In osteoblasts and osteocytes, IGF-I signaling is also activated in response to PS, leading to enhanced IGF production, IGF responsiveness, and inhibition of TNF-α-induced apoptosis, whereas inhibition of IGF-I abolishes loading-induced osteoblast proliferation (79–83). In osteoblasts, IGF-I synthesis is positively controlled by PTH *via* a cAMP-dependent mechanism (84). Interestingly, PTH was shown to enhance the PS-induced osteogenic response to PS, although this effect was found to be age dependent, indicating the complex interplay between MS, PS, and aging (85).

While mice overexpressing IGF-I reveal greater bone formation in response to PS than wild-type mice, knockout mice present with less periosteal bone formation as well as less trabecular bone volume, thickness, and density (82, 86). Similarly, GH has been found to modulate the levels of loading required to induce bone formation (87).

Inflammatory environments as found in patients exposed to MS can restrict osteocyte responses to PS, altering mechanotransduction mechanisms in bone (88). On the other hand, physiological PS prevents cytokine-induced osteoclast activation and bone loss exerting a protective role during inflammatory conditions and possibly MS (88, 89). MS, i.e., an inflammatory environment, could thus enhance osteocyte-to-osteoclast communication and osteoclastogenesis, whereas PS could be counteractive.

A further example is sustained physical conditioning that improves the performance of several allostatic mediators; for example, physically trained humans show a “trained HPA function” represented in a decreased HPA response under certain stress conditions (90–92). On the contrary, chronic strenuous exercise (CSE) can lead to overtraining, which is hallmarked by maladapted responses to excessive exercise without adequate rest, and perturbs multiple body systems (nervous, endocrine, and immune). CSE can increase basal glucocorticoid levels provoking mild hypercortisolism, diminishing the reactivity of adrenocorticotropic hormone and cortisol to CRH comparable to that of depressed persons. CSE also increases catecholamine levels, favoring a Th2 immunity profile, and inhibits long-term gonadal function (90, 93). To summarize, moderate PS provides health benefits, while CSE and overtraining can provoke biochemical and clinical abnormalities. This also influences bone quality, as already described in the “female athlete triad” (reproductive dysfunction, infertility, and osteoporosis) (90).

While current results point toward cross talk between PS and MS, further evidence for interaction, identification of dose–responses, and elucidation of molecular mechanisms is needed. Therefore, models of MS should initially be combined with existing animal models of PS, such as cyclic compression of caudal vertebrae, cyclic axial loading of the ulna or vibration platforms in rabbits, rats, or mice and later translated to human studies (94–98).

## Conclusion

In addition to comprehensive mechanobiological concepts showing the importance of PS in bone health and disease, compelling evidence has recently emerged that biochemical and psychoneuroendocrinological maladaptations caused by MS are not only also relevant for bone quality, but may furthermore considerably interact with PS. Furthermore, it is unclear how age-related risk factors interplay and/or whether they can synergistically impair bone health.

Based on the highlighted limitations of previous research as well as current gaps in our knowledge, we propose several new research avenues in humans and animals including (1) the investigation of different types of MS (traumatic/chronic/acute) as well as their molecular mechanisms and dose-dependent effects on bone deformation and structure, (2) the incorporation of physical activity in models of MS, (3) investigation of genetically modified animals for evaluation of mechanistic effects of PS in environmental conditions, (4) epigenetics, and (5) the investigation of aging within the aforementioned studies.

## Author Contributions

P-MW, MR, GK, and KW-K drafted, edited, and finalized the manuscript; approved the final version of manuscript; and agreed to be accountable for all aspects of the work.

## Conflict of Interest Statement

The authors declare that this mini review was written in the absence of commercial or financial relationships that could be interpreted as possible conflicts of interest.

## Abbreviations

ACTH, adrenocorticotropic hormone; AP-1, activator protein 1; BMD, bone mineral density; CRH, corticotropin-releasing hormone; CSE, chronic strenuous exercise; CT, computer tomography; GH, growth hormones; GHIH, growth hormone-inhibiting hormone; GHRH, growth hormone-releasing hormone; GR, glucocorticoid receptor; HPA, hypothalamic–pituitary–adrenal; HPG, hypothalamic–pituitary–gonadal; HPP, hypothalamic–pituitary–prolactin; HPS, hypothalamic–pituitary–somatotropic; HPT, hypothalamic–pituitary–thyroid; IGF-I, insulin-like growth factor-I; IL, interleukin; MS, mental stress; NF-κB, nuclear factor kappa B; PS, physical stress; PTH, parathyroid hormone; SAM, sympathetic–adrenal–medullary system; SNS, sympathetic–nervous system; TNF-α, tumor necrosis factor alpha.
